# Red Blood Cell Distribution Width as a Predictive Biomarker for Postoperative Infections in Children Who Underwent Cardiac Surgery: A Single-Center Retrospective Study

**DOI:** 10.7759/cureus.34051

**Published:** 2023-01-22

**Authors:** Nora Alruzouq, Sundos Almarshad, Khawla Almarshad, Tayf BinSheeha, Dhuha Alghamdi, Mohamed S Kabbani

**Affiliations:** 1 College of Medicine, King Saud Bin Abdulaziz University for Health Sciences, Riyadh, SAU; 2 Cardiac Sciences, King Abdulaziz Medical City, National Guard Hospital Health Affairs, Riyadh, SAU

**Keywords:** bloodstream infections, pediatric cardiac surgery outcomes, pediatric cardiac intensive care, red cell distribution width (rdw), ventilator-associated pneumonia, urinary tract infections, surgical site infection, sepsis, red blood cell distribution width, cardiac surgery

## Abstract

Background

It has been investigated that red blood cell distribution width (RDW) is associated with the clinical outcomes of patients following surgeries and is used as a prognostic biomarker for postsurgical complications. In this study, we aimed to assess the value of RDW as a predictor of postoperative complications in children after cardiac surgeries.

Methods

Three hundred fifty-five pediatric patients who underwent cardiac surgery between 2017 and 2018 were enrolled, and preoperative and postoperative RDW values were determined. Data collected included demographics; incidence of postsurgical complications, including sepsis, surgical site infections (SSIs), urinary tract infections (UTIs), and ventilator-associated pneumonia (VAP); length of hospital and pediatric cardiac intensive care unit (PCICU) stay; and ventilator duration.

Results

Among children who underwent cardiac surgery, 29 (8.2%) of the cases developed bloodstream infections (BSIs), while urinary tract infections (UTIs) were observed in 32 (9.0%) of the cases, and ventilator-associated pneumonia (VAP) was observed in 36 of the cases (10.1%). Of all cases, surgical site infections (SSIs) were reported in 13 patients (3.7%). Significantly higher postoperative RDW levels were observed on days three (p-value=0.028), five (p-value=0.041), and seven (p-value=0.042) in cases of BSI. For UTI cases, only preoperative RDW levels (p<0.001) and postoperative day three RDW levels (p<0.049) were significantly higher than their counterparts. VAP cases had significantly higher RDW levels pre-operatively (p-value=0.002), which was also observed in postoperative RDW levels on days three (p-value=0.033), five (p-value=0.031), and seven (p-value=0.021) in comparison to their analogs (p-values<0.05). Furthermore, a significant relationship was found between preoperative RDW and length of intensive care unit (ICU) stay (95% CI 0.685-3.221, p-value=0.003, R2=0.104) and duration of mechanical ventilation (95% CI 0.549-1.938, p-value=0.001, R2=0.102).

Conclusion

RDW is a significant factor in predicting complications in pediatric patients' post-cardiac surgeries, including BSI, UTI, and VAP, which would consequently anticipate patients' clinical state after cardiac procedures.

## Introduction

Red blood cell distribution width (RDW) reflects the size and variation of mature erythrocytes in peripheral blood, and it is routinely reported in the complete blood count panel with a normal range of 11.5%-14.5% [1]. An increase in RDW of more than 14.5% is suggestive of greater than normal variation in red blood cell (RBC) size. It is used in the differential diagnosis of different types of anemia [2]. Recently, numerous studies have shown a correlation between higher RDW and adverse outcomes, including sepsis, cardiovascular events, and cerebrovascular events including overall mortality [3-5].

Elevated RDW conferred 1.73 times increased risk of all-cause mortality and 2.49 times increased risk of cardiovascular disease (CVD)-specific mortality in adults. Some explained mechanisms are that oxidative stress and inflammation may play a role given that both can reduce RBC survival [6,7], leading to more mixed populations of RBCs in circulation, which thereby results in higher RDW levels that can be observed in conditions such as Down syndrome [8], poor pulmonary function [9], and dialysis [10]. Wang et al. found a correlation between RDW and renal cell carcinoma grade and stage at the time of diagnosis, defining advanced disease with an RDW cut-off value of 13.15% [11]. Similarly, Li et al. and Periša et al. concluded that the RDW cut-off levels of 14.15% and 14.5%, respectively, have been used as independent predictors of poor survival in ovarian cancer [12] and lymphocytic leukemia [13].

Most significant publications to date highlight the association between RDW values and coronary artery disease, heart failure, coronary artery ectasia, stroke, atrial peripheral occlusive artery disease, and fibrillation. Furthermore, RDW was suggested as a biomarker of complications in these patients [14]. The value of RDW as a prognostic predictor in pediatrics was also discussed. In a study including 404 pediatric intensive care unit (PICU) patients, RDW was linked to intensive care unit (ICU) morbidity and mortality [15].

Li et al. have established that RDW and C-reactive proteins might be independent predictive values of mortality in non-cardiac pediatric intensive care unit (PICU) setups, as abnormal changes in these values were associated with higher rates of mortality; however, the clinical reliability and potency of RDW have not yet been fully understood [16,17]. Another retrospective study explored the correlation between RDW values and prognosis or severity of the disease among newborns admitted with sepsis. Remarkable variabilities in RDW values were found between subsequent groups: the sepsis group had a mean RDW value of 16.59, the severe sepsis group had a mean RDW value of 18.88, and the septic shock group had a mean RDW value of 19.71. The study also showed a positive correlation between mortality and RDW values [18]. The levels of RDW were found to be remarkably high at the time of late-onset sepsis in infants, specifically in those infected with gram-negative bacteria [19]. There is a knowledge gap in measuring the RDW level as a prognosis predictor for pediatric sepsis.

Thus, further studies are required to determine the level of significance of RDW values as a predictive biomarker for postsurgical complications [20]. This study aims to assess the value of RDW as a predictor for bloodstream infections (BSI), ventilator-associated pneumonia (VAP), urinary tract infections (UTIs), and surgical site infections (SSIs) in children admitted to the pediatric cardiac intensive care unit (PCICU) after cardiac surgery.

## Materials and methods

This study was approved by King Abdullah International Research Medical Center with an approval number SP-20-261-R and was conducted in the PCICU of the Cardiac Center at King Abdulaziz Medical City (KAMC). A non-interventional retrospective cohort approach was used to measure the incidence of postsurgical infections in relation to RDW levels.

All pediatric patients aged 0 to 14 years old who underwent cardiac surgery between 2017 and 2018 were enrolled in this study. Patients on extracorporeal membrane oxygenation (ECMO) and those who were diagnosed with Down syndrome, severe anemia (Hb<7 g/dL), or known hematological diseases were excluded. The demographic characteristics, surgical records, clinical outcomes, and laboratory findings from the time of admission to discharge of the study subjects were collected by all the investigators through electronic charts (BestCare System, Seoul National University Hospital, Seoul, South Korea). The study was approved by the Institutional Review Board (IRB H-01-R-005). Due to the nature of the study, informed consent was waived.

The obtained data were analyzed within a 95% confidence interval using Statistical Package for Social Science (SPSS), version 23.0 (International Business Machines Corporation (IBM), New York, USA). Categorical variables were presented as frequencies and percentages, and continuous variables were presented as the mean ± standard deviation. Data were checked for normality using the Shapiro-Wilk test and were found to be non-normally distributed.

The relationship between RDW levels, ICU stay, and duration of mechanical ventilation was analyzed by a simple linear regression test. The relationship between RDW levels and BSI, UTI, and VAP was assessed by the Mann-Whitney U test. Lastly, the relationship between RDW levels and SSI was established by the Kruskal-Wallis H test.

## Results

A total of 355 pediatric cases were included, and among them 186 (52.4%) were male. The median weight was 5.64 kg, and the median age was six months. About 96.6% of cases had no syndromes, and 83.7% of cases had no previous infections at the time of admission. BSI was observed in 8.2% of cases, UTI was observed in 9.0% of cases, VAP was observed in 10.1% of cases, and SSI was observed in 3.7% of cases (Table 1). The mean mechanical ventilation duration was 5.7 ± 10.4 days, and the mean PCICU stay was 14.8 ± 24.1 days (Table 2).

**Table 1:** Distribution of all categorical variables (n=355). ECMO: extracorporeal membrane oxygenation, CPR: cardiopulmonary resuscitation, SSI: surgical site infections, UTI: urinary tract infections, VAP: ventilator-associated pneumonia, AKI: acute kidney injury, URTI: upper respiratory tract infection.

Variables	Attributes	N	%
Gender	Male	186	52.4
	Female	169	47.6
Syndrome	No syndrome	343	96.6
	Syndrome	12	3.4
Complications	No	186	52.4
	AKI	48	13.5
	Chylothorax	28	7.9
	Rhinovirus infection URTI	17	4.8
	Arrhythmia	15	4.2
	Pacemaker needed	8	2.3
	Need for CPR	5	1.4
	Need for ECMO	3	0.8
	Bleeding	3	0.8
	Other	42	11.8
SSI	No	324	91.3
	Suspected with culture	18	5.1
	Infected	13	3.7
Bloodstream infection	No	326	91.8
	Yes	29	8.2
Organism in blood	None	328	92.4
	Gram negative	17	4.8
	Gram positive	10	2.8
UTI	No	323	91.0
	Yes	32	9.0
Organism in urine	None	324	91.3
	Gram negative	24	6.8
	Gram positive	2	0.6
	Fungal	5	1.4
VAP	No	319	89.9
	Yes	36	10.1
Organism in VAP	None	320	90.1
	Gram negative	26	7.3
	Gram positive	8	2.3
	Fungal	1	0.3

**Table 2:** Distribution of continuous variables. MV: mechanical ventilation, NIMV: non-invasive mechanical ventilation, PCICU: pediatric cardiac intensive care unit, Hb: hemoglobin, WBC: white blood cells, RDW: red cell distribution width, D3: day 3, D5: day 5, D7: day 7.

Variables	N	Mean	Standard deviation	Median
Weight	353	9.3612	9.51125	5.6400
Age	353	24.1900	37.61422	6.0000
MV duration	317	5.6782	10.39218	2.0000
NIMV duration	202	5.4653	27.67026	5.0000
PCICU stay	352	14.8040	24.14967	8.0000
Hospital stay	355	24.8901	30.34122	18.0000
Preoperative Hb	351	131.8376	23.97461	128.0000
Preoperative WBC	350	10.4515	4.04239	9.9500
Preoperative RDW	350	15.7937	2.30197	15.5000
Postoperative Hb	352	123.3929	22.84560	120.5000
Postoperative WBC	353	12.6527	7.54898	11.7000
Postoperative RDW (D3)	351	15.9843	2.06776	15.6000
Postoperative RDW (D5)	296	16.0821	2.09779	15.7000
Postoperative RDW (D7)	250	15.9608	2.11597	15.5000

The relationship between RDW levels and BSI, UTI, VAP, and SSI was assessed. Significantly higher postoperative RDW levels were seen in cases with bloodstream infections. The p-value for postoperative day three RDW was 0.028, the p-value for postoperative day five RDW was 0.041, and the p-value for postoperative day seven RDW was 0.042. Patients who developed VAP had higher RDW levels both pre- and postoperatively (p-value<0.050). For UTI cases, only preoperative RDW levels (p<0.001) and postoperative day three RDW (p-value=0.049) were significantly higher. For patients with SSI, RDW levels preoperative and postoperative on days three and five were found to be significantly higher (p-value<0.050) (Tables 3, 4).

**Table 3:** Mean RDW levels in infected and non-infected patients. RDW: red cell distribution width, D3: day 3, D5: day 5, D7: day 7, BSI: bloodstream infections, UTI: urinary tract infections, VAP: ventilator-associated pneumonia, SSI: surgical site infections.

Infected					Non-infected			
BSI		UTI	VAP	SSI	BSI	UTI	VAP	SSI
Mean preoperative RDW	16.47 ± 2.11	17.17 ± 1.79	16.80 ± 2.04	16.68 ± 2.03	15.74 ± 2.31	15.66 ± 2.30	15.68 ± 2.31	15.66 ± 2.31
Mean postoperative RDW (D3)	16.52 ± 1.64	16.55 ± 2.09	16.64 ± 2.50	17.44 ± 2.03	15.94 ± 2.10	15.93 ± 2.06	15.91 ± 2.01	15.89 ± 2.07
Mean postoperative RDW (D5)	16.66 ± 1.66	16.35 ± 2.15	16.89 ± 2.70	17.04 ± 1.66	16.04 ± 2.12	16.05 ± 2.09	15.98 ± 1.99	16.01 ± 2.14
Mean postoperative RDW (D7)	16.79 ± 2.05	15.98 ± 2.02	16.85 ± 2.76	16.54 ± 1.80	15.89 ± 2.11	15.96 ± 2.13	15.83 ± 1.98	15.92 ± 2.19

**Table 4:** Relationship between RDW levels and BSI, UTI, VAP, and SSI. RDW: red cell distribution width, D3: day 3, D5: day 5, D7: day 7, BSI: bloodstream infections, UTI: urinary tract infections, VAP: ventilator-associated pneumonia, SSI: surgical site infections.

Infected								
Mean preoperative RDW		p-value	Mean postoperative RDW (D3)	p-value	Mean postoperative RDW (D5)	p-value	Mean postoperative RDW (D7)	p-value
BSI	16.47 ± 2.11	0.077	16.52 ± 1.64	0.028	16.66 ± 1.66	0.041	16.79 ± 2.05	0.042
UTI	17.17 ± 1.79	0.001	16.55 ± 2.09	0.049	16.35 ± 2.15	0.481	15.98 ± 2.02	0.921
VAP	16.80 ± 2.04	0.002	16.64 ± 2.50	0.033	16.89 ± 2.70	0.031	16.85 ± 2.76	0.021
SSI	16.68 ± 2.03	0.001	17.44 ± 2.03	0.001	17.04 ± 1.66	0.014	16.54 ± 1.80	0.116

Simple linear regression analysis showed that there is a significant relationship between preoperative RDW and length of ICU stay (95% CI 0.685-3.221, p-value=0.003, R2=0.104).

Similarly, high preoperative RDW levels significantly correlated with longer mechanical ventilation duration (95% CI 0.549-1.938, p-value=0.001, R2=0.102). However, the length of ICU stay and mechanical ventilation was not related to postoperative RDW levels (Tables 5, 6). Finally, preoperative and postoperative RDW levels and their association with mortality were not significant (Table 7).

**Table 5:** Relationship between RDW levels and ICU stay. RDW: red cell distribution width, D3: day 3, D5: day 5, D7: day 7, ICU: intensive care unit.

Variables	Beta	t	Sig.	95% Confidence interval for B	
				Lower bound	Upper bound
Preoperative RDW	0.234	3.034	0.003	0.685	3.221
Postoperative RDW (D3)	−0.068	−0.553	0.581	−3.037	1.706
Postoperative RDW (D5)	0.099	0.564	0.573	−2.334	4.208
Postoperative RDW (D7)	0.090	0.667	0.505	−1.676	3.392

**Table 6:** Relationship between RDW levels and duration of mechanical ventilation. RDW: red cell distribution width, D3: day 3, D5: day 5, D7: day 7.

Variables	Beta	t	Sig.	95% Confidence interval for B	
				Lower bound	Upper bound
Preoperative RDW	0.282	3.530	0.001	0.549	1.938
Postoperative RDW (D3)	−0.148	−1.150	0.251	−2.036	0.536
Postoperative RDW (D5)	−0.117	−0.605	0.546	−2.446	1.298
Postoperative RDW (D7)	0.294	1.886	0.061	−0.066	2.992

**Table 7:** Relationship between RDW levels and mortality. RDW: red cell distribution width, D3: day 3, D5: day 5, D7: day 7.

Alive		Death	p-value
Preoperative RDW	15.79 ± 2.32	15.98 ± 0.81	0.463
Postoperative RDW (D3)	15.98 ± 2.08	16.00 ± 1.03	0.639
Postoperative RDW (D5)	16.08 ± 2.11	16.16 ± 1.07	0.560
Postoperative RDW (D7)	15.95 ± 2.13	16.60 ± 1.08	0.217

## Discussion

There is emerging evidence of the value of RDW levels in adult patients with heart failure, myocardial infarction, paroxysmal atrial fibrillation, pulmonary embolism, and chronic obstructive pulmonary disease (COPD) [21]. Several studies have shown that a high RDW can be used as a strong, independent predictor of increased mortality and morbidity [22,23]. It has, therefore, become a valuable tool used to assess patients with cardiac diseases for any possible complications. However, most of the published research has only concentrated on the value of RDW levels in adults undergoing cardiac surgery and critically ill children. 

There is paucity in the literature considering the value of RDW as a biomarker predictor for postoperative infections, particularly in children. To our knowledge, this is the first report nationally discussing the prognostic significance of RDW, at both pre- and postoperative levels, in predicting complications in pediatric patients undergoing cardiac surgery. RDW is believed to be a significant marker of cardiovascular illnesses due to the contribution of inflammation and oxidative stress to the progression of cardiovascular disease. Although the exact mechanism of this implication has not been fully identified yet, hypotheses relating to oxidative stress and inflammatory responses have led the way in several examples of relevant research. Erythrocytes are more susceptible to the effects of oxidative damage due to their greater antioxidant capability. Oxidative stress and chronic inflammation reduce erythropoietin production and exacerbate the destruction process of erythrocytes, resulting in inefficient red blood cell formation, damage to erythrocytes, a reduction in the life cycle of available erythrocytes, and the entry of the immature cells into the systemic circulation, thus increasing levels of RDW [24].

In this study, pre- and post-operative RDW levels were assessed and found to be higher preoperatively in patients who developed UTIs, particularly with gram-negative bacteria (6.8%) after surgery. Although, in this group, there was no significant elevation in RDW values after the surgery. Fungal VAP was found to be of higher incidence in patients who had elevated RDW levels before and after surgery. Controversially, Sachdev et al. reported no association between RDW levels at admission and the occurrence of infections during hospitalization [25].

Moreover, in this present study, increased preoperative RDW levels were found to be associated with an increased length of PCICU stays. This impression is strengthened by the same observation by Sachdev et al., who clearly stated that higher RDW levels at admission were correlated with prolonged PICU stays in critically ill children [25,26]. Wang et al. also looked at adult patients who underwent coronary artery bypass grafts and found that those with higher RDW levels had prolonged hospital stays [27]. It was also observed in this current study that patients with high RDW levels upon admission have had prolonged mechanical ventilation support during their stay in the PCICU. However, the association between preoperative RDW levels and the duration of mechanical ventilation was not found in the literature. Several studies on both pediatrics and adults have reported that persistently high RDW is associated with a higher in-hospital mortality rate [23,27]. However, this association was not observed in this study, as both pre- and post-operative RDW levels were not a significant factor in determining in-hospital mortality in children who underwent cardiac surgery. A strong link was also found between high RDW readings on the third, fifth, and seventh days post-surgery and the presence of BSI. This is in keeping with the results reported by Ahmed et al., who found that RDW levels were significantly higher in neonates who developed sepsis during their ICU stay [28].

In addition, the association between elevated RDW after surgery and the presence of SSI was found to be significant in this current study. This finding was also observed by Kumar et al., who looked at children’s recovery after correction of Tetralogy of Fallot and found that patients with increased postoperative RDW levels had more frequent SSIs [29].

Limitations 

There are a few limitations to this study. First, it was a single-centered study. Second, most of the subjects of interest underwent multiple different non-cardiac procedures; consequently, the studied patients were unified by including only those who underwent cardiac surgeries that were included in the risk-adjusted classification for the congenital heart surgery category (RACHS-1) [30].

## Conclusions

In conclusion, the present study has discussed the use of RDW as a valuable marker for predicting complications in pediatric patients following cardiac surgeries, including bloodstream infections, UTI, and VAP. Higher RDW values pre- and postoperatively were recognized in patients who developed VAP, UTI, and SSI. However, in relation to BSI, high RDW levels were only observed postoperatively. Higher preoperative RDW levels were found to correlate with longer ICU stays and mechanical ventilation duration. The results reached in this study would consequently aid in anticipating the patients’ clinical state after cardiac procedures by investigating a single biomarker. Although further extensive research is required to assess the prognostic value of RDW and to reliably and indisputably correlate RDW with certain complications, this parameter is considered to be a fairly significant tool to be contemplated in evaluating morbidity, prognosis, and estimation of the length of hospital stay in pediatric patients with a recent history of cardiac surgeries.
